# Supplementary material for: Rapid drug increase and early onset of levodopa-induced dyskinesia in Parkinson’s disease
Source: PLoS One. 2020 Aug 20;15(8):e0237472. doi: 10.1371/journal.pone.0237472 (PMC7444533; doi:10.1371/journal.pone.0237472)
Supplement: Attachment — Submitted filename: Response to Reviewers.doc [file pone.0237472.s001.doc]

**Response to Reviewers**

**PLOS ONE manuscript ID#: PONE-D-20-13495
Title: Rapid drug increase and early onset of levodopa-induced dyskinesia in Parkinson’s disease**

**Reviewer #1**

- Could the authors precise how they included the subjects in their study: did they select patients who were known to have early/middle and late LID onset or did they included consecutive patients followed in the different centers and then, classified them in 3 different groups, a posteriori?

Answer) This study was conducted using medical record and study subjects were retrospectively selected according to the study criteria. Now we clarify and rewrite the Method section in revised manuscript.

- Could the authors argue the reason for which they excluded patients with very early LID? It would have been interesting to include them as they usually reflect severe dopamine depletion.

Answer) Importance of this study is on the method for assessment of drug dose. The rate of dose increase was calculated as (change in dose / treatment duration), therefore relatively short duration of treatment may make the rate of dose increase overestimated. To reduce this chance, we selected patients who encountered LID at least two years after initiation of levodopa. We declare this in Method section.

- Which criteria did they use to consider that patients were at LID onset? Did they took the medical observation into consideration? The (MDS)-UPDRS part IV for complications of treatment? The Marconi LID scale? Patients questionnaire? We know that slight dyskinesia may occur early in disease evolution but patients are lot always aware of them and are not always able to recognize them as dyskinesia. It may be, thus, a limit to define the precise onset of LID.

Answer) Because this study focused on the time to onset of dyskinesia, presence or absence of LID is the only data needed. Scales for dyskinesia is for assessment of severity of LID were not used in this study. As reviewer comment, many patients are not aware of their dyskinesia. Therefore, we defined the onset of LID based on clinicians’ observation. But when patients reported onset of dyskinesia and clinician also observed the dyskinesia, we accepted the day that patients indicated. Now we describe this content in Method section of revised manuscript.

- In Table 1 the authors reported the number of patients with treatment other than levodopa: was it the treatment, patients had, at time of LID onset?

Answer) Drug other than levodopa was assessed at the time of LID onset. In revised manuscript, we add this information in Table 1 and Result section.

- In the results section the authors mentioned that female subjects were at higher risk of early LID but they wrote that male subjects were at higher risk in the abstract: please correct.

Answer) We thank for reviewer’s correction. We correct the abstract.

- The authors associated the risk of LID onset with the rapid rate of levodopa /LED increase. However, it is known that LID are also associated with pulsatile post-synaptic dopaminergic stimulation.

- If data is available, and in order to help interpreting the results of the study, the authors could study and report the way levodopa was prescribed and whether the increase in levodopa dose or LED was associated with an increase in levodopa/LED unitary dose (which may enhance pulsatile answer and promote LID) or whether the increase was associated an increase in the number of daily intakes (a more continuous dopaminergic administration, which may delay the onset of LID). If the authors could add these data, they could develop more precisely the discussion on the pathophysiology of LID and the way to manage PD patients to avoid the occurrence of LID, even in severely depleted patients.

Answer) We selected patients who was treated with levodopa initially, and almost all the patients took levodopa three time a day. And, no patient of this study was treated with continuous intestinal levodopa, subcutaneous apomorphine, rotigotine patch, or deep brain stimulation. Therefore, we think that influence of pulsatile dopaminergic stimulation on onset of LID cannot be explored using this study data and subanalysis suggested by reviewer seems to be impossible in this data

**Reviewer #2**

Major comments
MATERIALS AND METHODS
Subjects :
- Authors chose to perform the analyses with de novo PD patients. It is well known that some PD patients are in fact misdiagnosed (Coarelli et al, J Neurol Sci, 2019 ; Hustad E, Aasly JO. Front Neurol. 2020). How did they ensure that these patients were truly PD patients and not parkinsonian patients from other degenerative disorders such as PSP, MSA,… Which clinical criteria did they use? Did they check these criteria at the beginning of the « PD » disease but also during the follow-up of these patients? It seems strange that authors did not observe other parkinsonian disorders due to initial misdiagnosis, even if they are experts in movement disorders.

Answer) We did not enroll study patients consecutively, but reviewed medical record and selected proper patients for study. During patient selection, other parkinsonian patients such as atypical parkinsonism, dementia with Lewy bodies, frontotemporal dementia, or unspecified disorders were excluded. Now we describe the process of patient selection in detail in Method section of revised manuscript.

- Did the authors use a score of dyskinesia such as the UDysRS?

Answer) This study focused on the time to onset of dyskinesia, so scales for assessment of severity of LID were not used.

- In the sentence « Data of patients with… drug dose », the « for » seems not appropriate or something is missing.

Answer) According to reviewer’s comment, we revise the sentence.

- How was performed the motor UPDRS by authors? Was it applied in the OFF state or in the ON state (or intermediate state)? How long after the last medication did the UPDRS have been carried out? Please detail the assessment of the UPDRS score.

Answer) UPDRS score rated at de novo status (just before initiation of levodopa) was used in this study. Now we add this content in Method section of revised manuscript.

RESULTS
- Demographic characteristics : it is probably more accurate to rename this part « Demographic and clinical characteristics »

Answer) According to reviewer’s suggestion, we now rename the title of the paragraph and table 1.

- The UPDRS motor score was available for only 89 out of 150 patients. Could you precise how many UPDRS motor scores were available in each group (E, M, L). Moreover, in which condition (On? OFF? intermediate?) were assessed these scores?

Answer) As per reviewer’s suggest, we now present the number of patients of each group who had been assessed with UPDRS motor score and modified Hoehn and Yahr stage (footnote of Table 1). The used score rated at only drug-naïve status.

- The UPDRS is relatively elevated at initiation of Levodopa and/or dopaminergic agonists. It seems that patients were not sufficiently treated. Could you please justify this point?

Answer) First, the UPDRS score was rated at de novo status but not in treatment period. The mean UPDRS motor score at de novo status ranges 20 to 25 in most studies. Moreover, we selected patients who initiated medical treatment with levodopa, so patients with mild symptom who was suitable for dopamine agonist or MAO-B inhibitor monotherapy may be excluded from this study.

- Entacapone increases the occurrence of dyskinesia (Ahn et al., J Clin Neurol. 2007 ; Trenkwalder et al., Neurology. 2019). Did authors observe any difference in dyskinesia occurrence between patients who took entacapone and those who did not take it? Did they look for a difference in each group (E, M, L)?

Answer) The numbers of patients treated with entacapone are already presented in Table 1. There was no difference in number of patients treated with entacapone among E, M, and L groups.

- In the « Risk factor for early LID » section, second line, authors declared that there was a correlation between time to onset of LID and LED but the p value is 0.066 which is above 0.05… (you should also correct the sentence related with this result in the Discussion section)
Answer) As per reviewer’s comment, we revised the sentence for the data.

DISCUSSION
Concerning the sentences from «However the present results… » to « … acts as a trigger for onset » :
It is well established that the occurrence of LID is secondary both to the dopaminergic denervation AND the levodopa administration. Without one of this factor, LID could not appear. LID occur in patients (or animals) taking Levodopa but only in the presence of dopaminergic degeneration.
Answer) In the paragraph, we focused on the role of levodopa dose. In the clinical setting, levodopa dose was affected by severity of dopaminergic denervation, therefore it is unclear whether levodopa dose is an independent factor for LID or higher levodopa dose is just a consequence of severe dopamine depletion. We discussed this point in this paragraph. Now we revise the paragraph to make it less confusing.

Additionally, as reviewer commented, both dopaminergic denervation and administration of dopaminergic drug are essential for onset of LID. But, Tagasaki et al. (Neuropharmacology 2005) showed that therapeutic dose of levodopa induced LID in DAT blocker-treated squirrel monkey without dopaminergic denervation, and Pearce et al. (Psycholpharmacology 2001) demonstrated that higher dose of levodopa induced LID in dose dependent manner in normal macaque monkey. Therefore, we think that the role of levodopa on LID should be reconsidered.

Minor comments
- Replace « Table 2 » by « Table 1 » in the « Drug » section of the Results.
- Insert « Table 2 » at the end of the sentence « Multivariable linear regression models … were associated with early onset of LID ». Same remark for the next sentence.

Answer) We appreciate reviewer’s correction. Now we edit that according to the comment.
