# Supplementary material for: Rapid drug increase and early onset of levodopa-induced dyskinesia in Parkinson’s disease
Source: PLoS One. 2020 Aug 20;15(8):e0237472. doi: 10.1371/journal.pone.0237472 (PMC7444533; doi:10.1371/journal.pone.0237472)
Supplement: Attachment — Submitted filename: Response to Reviewers_R2.doc [file pone.0237472.s002.doc]

**Response to Reviewers**

**PLOS ONE manuscript ID#: PONE-D-20-13495R1
Title: Rapid drug increase and early onset of levodopa-induced dyskinesia in Parkinson’s disease**

**Reviewer #1**

The authors did not add any comment on pharmacokinetics and the role of pulsatile administration of levodopa in sensitization and in the development of motor complications (see Nutt J, 1995, Clin Exp Pharmacol Physiol, Nutt J, 2007, Movement Disorders Journal, Sharma et al, 2015 Biomed Pharmacother for example). Most of the patients were taking treatment 3 times a day, as noted by the authors in the answers to comments. It did not seem to be different across groups. This would argue against the idea that high unitary doses of levodopa increase the risk of LID, whereas the rate of increase seem to be more important.
They could perhaps add this comment in the limits of the study section.

Answer) Now, we add the contents about the way levodopa was initiated and increased in Method section (lines 104-106 in revised manuscript). In this study, levodopa was usually prescribed three times a day with equal dose, and we increased the levodopa by 50mg per dose (150mg a day) when dose increased was needed. As we answered in previous response to review, there was no change in the number of daily intakes of levodopa during dose increase and there was no difference in number of daily intakes among patients. Therefore, we think that issue for the continuous dopaminergic stimulation is not applicable to this study.

**Reviewer #2**

- Please use « levodopa » or « Levodopa » but authors should homogeneize this in all the manuscript
Answer) Now we use “levodopa” in this revision.

- Lines 74-75, replace « Among them, we selected patients who medical treatment initiated with levodopa » by « Among them, we selected patients who initiated medical treatment with levodopa »
- Lines 82-84 : I think authors should rewrite the sentence « When patient reported symptom suggesting LID or dyskinetic movement was observed in the clinic, clinicians have been recording the information for LID » ; « When patient reported symptom suggesting LID or dyskinetic movement was observed in the clinic, clinicians recorded the information for LID
Answer) We revise the sentences according to the suggestion.

- Line 89 : « developed within 2 year … » ; add a « s » for years : « developed within 2 years … »
- Line 94 : please put « de novo » in italic
- Table 1 : « P-value » should be replaced by « p-value »

Answer) We correct the errors.
